# How to survey classical swine fever in wild boar (*Sus scrofa*) after the completion of oral vaccination? Chasing away the ghost of infection at different spatial scales

**DOI:** 10.1186/s13567-015-0289-6

**Published:** 2016-01-25

**Authors:** Thibault Saubusse, Jean-Daniel Masson, Mireille Le Dimma, David Abrial, Clara Marcé, Regine Martin-Schaller, Anne Dupire, Marie-Frédérique Le Potier, Sophie Rossi

**Affiliations:** ONCFS, Office National de la Chasse et de la Faune Sauvage, Unité Sanitaire de la Faune, Micropolis, la Bérardie, Belle Aureille, 05000 Gap, France; Anses, Laboratoire de Ploufragan/Plouzané, Unité Virologie Immunologie Porcines, BP53, 22440 Ploufragan, France; INRA, Unité d’Epidémiologie Animale, Theix, 63122 Saint-Genès-Champanelle, France; Direction générale de l’alimentation, Bureau de la santé animale, Paris, France; Direction départementale de la protection des populations du Bas-Rhin, Strasbourg, France; Direction départementale de la protection des populations de la Moselle, Metz, France

## Abstract

Oral mass vaccination (OMV) is considered as an efficient strategy for controlling classical swine fever (CSF) in wild boar. After the completion of vaccination, the presence of antibodies in 6–12 month-old hunted wild boars was expected to reflect a recent CSF circulation. Nevertheless, antibodies could also correspond to the long-lasting of maternal antibodies. This paper relates an experience of surveillance which lasted 4 years after the completion of OMV in a formerly vaccinated area, in north-eastern France (2010–2014). First, we conducted a retrospective analysis of the serological data collected in 6–12 month-old hunted wild boars from 2010 up to 2013, using a spatial Bayesian model accounting for hunting data autocorrelation and heterogeneity. At the level of the whole area, seroprevalence in juvenile boars decreased from 28% in 2010–2011 down to 1% in 2012–2013, but remained locally high (above 5%). The model revealed the existence of one particular seroprevalence hot-spot where a longitudinal survey of marked animals was conducted in 2013–2014, for deciphering the origin of antibodies. Eleven out of 107 captured piglets were seropositive when 3–4 months-old, but their antibody titres progressively decreased until 6–7 months of age. These results suggest piglets were carrying maternal antibodies, few of them carrying maternal antibodies lasting until the hunting season. Our study shows that OMV may generate confusion in the CSF surveillance several years after the completion of vaccination. We recommend using quantitative serological tools, hunting data modelling and capture approaches for better interpreting serological results after vaccination completion. Surveillance perspectives are further discussed.

**Electronic supplementary material:**

The online version of this article (doi:10.1186/s13567-015-0289-6) contains supplementary material, which is available to authorized users.

## Introduction

Classical Swine Fever (CSF) is one of the diseases entailing strong economic impact on the pig industry in the European Communities [1]. Eradicated from domestic pigs in Western Europe, CSF has remained endemic in some populations of wild boar (*Sus scrofa*) for more than 20 years. Thus, free ranging populations of European wild boar are regarded as potential reservoirs of CSF [2, 3] and their monitoring and management is compulsory in the European Communities (Directive 2001/89/EC). Oral immunisation has appeared as an effective management strategy for controlling CSF outbreaks in wild boar in contrast with conventional control measures (e.g., increase of hunting pressure and hunting of young animals) [4–6]. The live C-strain, an attenuated CSF virus, has been repeatedly delivered by mean of baits to wild boars pre-baited on feeding stations [4]. In facilities, a satisfying level of neutralizing antibodies, which may last lifelong, was observed already after a single vaccination dose was orally administrated [7]. Repeated vaccination treatments (in facilities) increased the individual concentration of neutralizing antibodies [8] and increased high herd immunity in natural populations (such as detailed in [5] or [6]). Nevertheless, re-emergence of CSF was sometimes reported after long periods of apparent remission during which the disease was supposed to be eradicated [3], which has pinpointed the importance of maintaining the monitoring after the implementation of oral mass vaccination (OMV).

In the absence of vaccination, both direct observation of infection (through RT-PCR and virus isolation) and indirect through the detection of antibodies in young wild boars are good indicators of the recent circulation of CSF. Seroprevalence in juveniles from 6 to 12 months-old is particularly useful when using hunting data since the viremia is generally short while wild boar recovering from infection will keep antibodies for life [3, 9]. However, this indicator is compromised during OMV based on an attenuated live virus not modified since diagnostic methods cannot differentiate antibodies targeting the wild strain from antibodies targeting the vaccine strain [10]. During the conduction of OMV, the surveillance is therefore only based on virus detection results, but viroprevalence is very low [3, 5, 11] and RT-PCR methods have to be adapted to distinguish between vaccine and wild strains to avoid inconclusive results in the presence of the vaccine-strain [12, 13]. Due to the confusing effect of OMV, the absence of viral detection for a long period (i.e., up to 1 year) is recommended before the completion of OMV [3]. During the 3 years after the completion of OMV, the examination of antibodies in 6–12 month-old wild boars is recommended for monitoring the risk of CSF virus persistence or re-emergence [14], with a particular attention given to the hot spot areas exhibiting seroprevalence above 5% [3]. Nevertheless, until now no study has detailed the post-vaccination monitoring of serological responses. In particular no study has yet discussed the confusing effect of repeated vaccination treatments on the performance of a surveillance design based on serological results. Different mechanisms may complicate a monitoring based on seroprevalence in 6–12 month-old wild boar as an indicator of recent CSF infection. Maternal antibodies transmitted by immune sow to their offspring generally do not persist more than 3 months post-partum [15] while juvenile wild boar are generally not hunted before 6 months of age [3, 14]. However, 70% of adult wild boars remained immune during the implementation of OMV [6, 16] and it has been experimentally demonstrated that maternal antibodies may persist up to 1 year in some piglets when the level of neutralizing antibodies is very high in sows [17]. Even though this phenomenon is not very frequent in natural conditions (unknown percentage), it is reasonable to assess long-lasting maternal antibodies may happen at the level of a population comprising at least 20 000 wild boars. The vaccine strains rarely survive for more than a few days at room temperature [18], so wild boar immunization with baits remaining in the environment after OMV was stopped seems a less probable scenario. Nevertheless, we did not fully reject that hypothesis since millions of baits were delivered at a large scale and exceptional survival of vaccine cannot be excluded [16]. Thus antibodies in 6–12 month-old hunted wild boar after the completion of OMV may finally correspond to three situations: (1) wild boar infection and recovery during the current year, (2) immune sows having transmitted a high amount of antibodies to their offspring through the colostrum leading to exceptional seropositive results in piglets over 6 months of age, or (3) piglets having eaten a viable vaccine-bait remaining in the environment after vaccination was stopped.

In the present study we aimed at improving CSF surveillance in wild boar after the completion of OMV. We focused our study in the Vosges du Nord area, north-eastern France, where two consecutive CSF outbreaks had been previously described during the 1990s and the 2000s [19–21] and where OMV using C-strain live-vaccine had been implemented from August 2004 up to June 2010 [6]. We adopted a two step surveillance approach combining two spatial scales of data collection. First, we fitted a “disease” mapping model of the serological data collected in 6–12 month-old hunted wild boars during the 3 years following the completion of OMV for identifying the hot spots of seroprevalence in that age class. Secondly, repeated captures of wild boar were organized within the identified hot spot areas for examining the individual kinetics of neutralizing antibodies and CSF infection in 2–18 month juvenile wild boar and in adult sows (i.e., over 24 months of age) from the same social groups. We were able to discuss the origin of antibodies in juvenile wild boars after the completion of OMV and to propose ad hoc perspectives for CSF surveillance.

## Materials and methods

### Retrospective study on hunted wild boars

#### Study area

**Figure 1:**
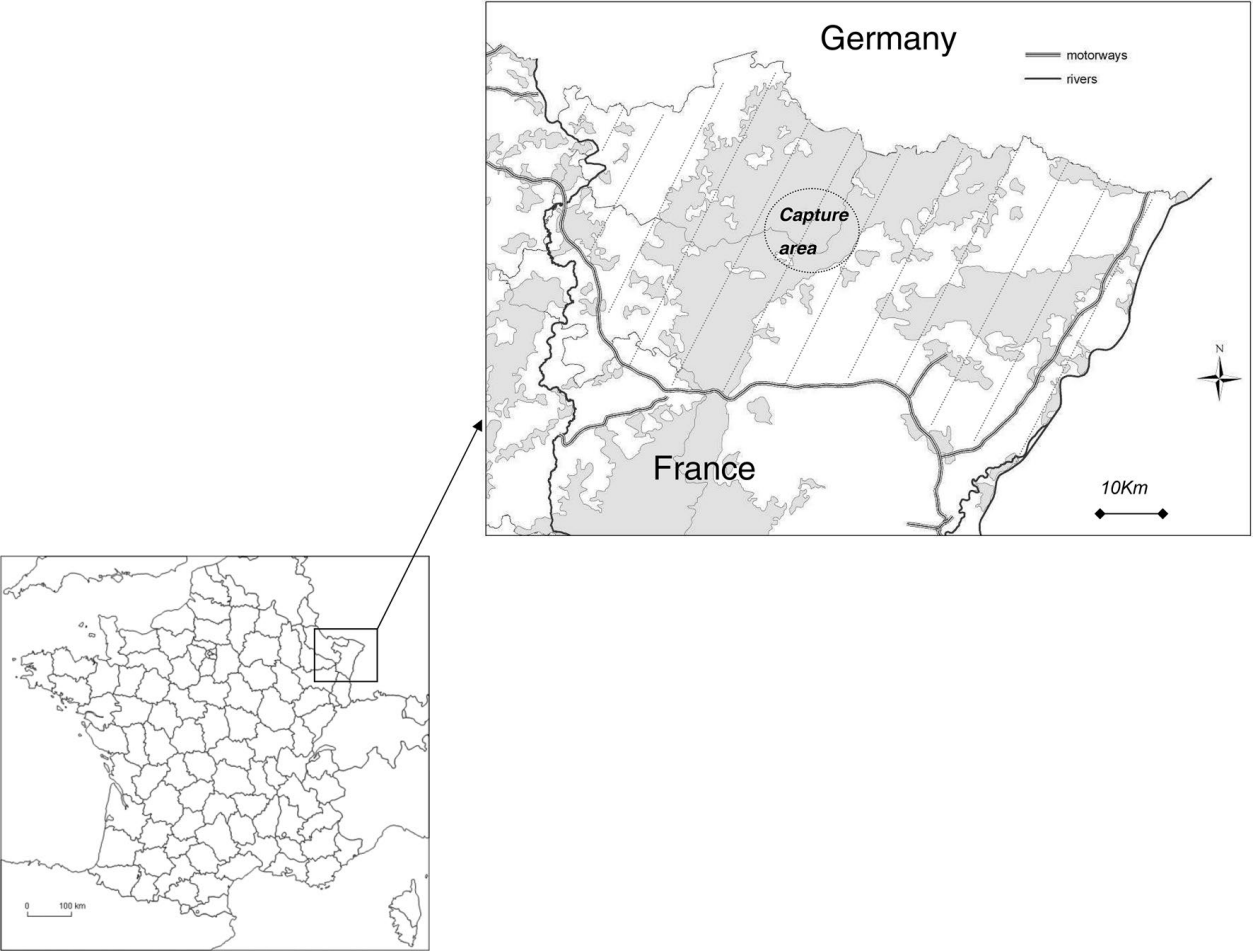
**Study area.** The formerly vaccinated area of the Vosges du Nord is hatched and the grey areas correspond to the forest cover.

The Vosges du Nord area is located within the Moselle and the Bas-Rhin administrative departments, north-eastern France (48°50N and 7°30E) [19, 20]. The study area covers 3000 km^2^ comprising 1200 km^2^ of forests and is uninterrupted with the Palatinate forest through Germany (Figure 1). CSF virus has been reported in either hunted or wild boars found dead from April 2003 up to May 2007 [6]. Vaccination was implemented from August 2004 according to the process recommended by Kaden et al. [4] using the Riems C- live-vaccine strain included in baits according to the field process detailed by Calenge and Rossi [16]. No virus positive wild boars had been observed since May 2007, the completion of the vaccination strategy was thus adopted by June 2010 (i.e., 3 years after the last viropositive result). Exhaustive monitoring of hunted and wild boars found dead was requested by the French ministry of agriculture from July 2010 up to October 2013, following the recommendations from European and National food safety agencies [3, 22].

#### Source of diagnostic samples

In the study area, every wild boar shot or found dead had been examined for serological and virological detection during the 3 years after the completion of vaccination, i.e., from July 2010 up to June 2013 [23, 24]. We more particularly focused on the occurrence of seropositivity in 6–12 month-old wild boars shot by hunters after the stop of OMV, i.e., between September 2010 up to June 2013. The data set was split into three time periods corresponding to the three successive generations of piglets born after vaccination (2010–2011, 2011–2012, 2012–2013). Each year was defined from October of the year “N” to June of the following year “N + 1”, because during that period animals less than 12 months-old were easily distinguished from older individuals according to their body mass and shape [19], while in the summer (July–August N + 1) a confusion might happen between animals more or less than 12 months-old [16].

#### Diagnosis

Detection of antibodies to CSFV was carried out using commercially available ELISA kits (Idexx CSF Ab) according to the manufacturer’s instructions by two local laboratories. All the sera with positive or doubtful results using ELISA collected from December 2012 up to June 2013 in young hunted wild boars were subsequently analysed using a differential virus neutralization test (VNT) in order to confirm the specificity of the ELISA results and to assess the titre of neutralizing antibodies. Specific neutralizing antibodies (NAb) against the CSFV Bas-Rhin strain versus the Aveyron strain of the border disease (BD) virus (Pestivirus of small ruminants) were assayed using the VNT on PK15 cells according to the OIE’s manual of diagnostic tests [25] by the National Reference Laboratory.

#### “Disease” risk mapping

“Disease” risk mapping for each time period was based on the serological results collected in the Vosges du Nord area through hunting at the level of 331 French administrative territories (village or town) that we call hereafter municipalities. The serological status was likely to be similar between wild boars inhabiting the same municipality by sharing the same antigenic background (i.e., virus circulation or vaccination treatment) and even belonging to the same family groups (and maternal antibody source). The serological status was also likely to be similar between wild boars inhabiting neighbouring municipalities, either because CSF is a contagious disease or because individual wild boars may be shot in a given municipality while the largest part of its home range may be included in a neighbouring one [26]. The occurrence of similar serological status within one municipality generates “heterogeneity”, while the occurrence of similar serological results between neighbouring municipalities generates “autocorrelation”. The geographical representations of raw seroprevalence (without data modelling) at the scale of administrative units could lead to misinterpretations of the observed clusters and consecutive inaccurate management decisions [27]. In order to avoid these problems, we took into account the probable heterogeneity and autocorrelation of serological results using hierarchical spatial Bayesian models [28, 29] (model detailed in Additional file 1). Data were encoded as “0” and “1” depending on their ELISA results; only positive or negative results were considered (i.e., inconclusive or doubtful results were removed). Seroprevalence per municipality was modelled year by year according to a hierarchical Bayesian approach proposed by Abrial et al. [30]. The probability to observe seropositive results in a given municipality “i” was modelled according to a Poisson distribution. The variation of data was modelled according to a local spatial component (*U*_*i*_) accounting for seroprevalence similarities between neighbour municipalities (i.e., spatially structured variation or autocorrelation) and a global one (*H*_*i*_) accounting for seroprevalence unstructured heterogeneity between municipalities. The conservation of the spatial pattern from year–year was also tested by considering the average seroprevalence predicted by the model retained for the previous year as a potential factor of seroprevalence within the model for the current year (*Risk*_*i*_). We retained for each year the most parsimonious model having the smaller Deviance Information Criterion (DIC; [31]). Simulations were calculated using the BRugs package [32], which constitutes the interface between the software OpenBugs and the R statistical environment (R Development Core Team [33]). Mappings were performed using QGIS (Quantum GIS Development Team, 2009 [34]).

### Capture-mark-recapture study

#### Capture and sampling process

In order to maximize the chances of capturing seropositive piglets, we targeted the areas exhibiting the highest seroprevalence in young wild boars (i.e., the hot spot municipalities that were identified by the hierarchical spatial Bayesian models). The capture area spread over 3000 hectares mainly comprising the municipality of Baerenthal and the neighbour ones (Figure 1). Physical captures were performed from the 2^nd^ of July up to the 30^th^ of August 2013. Twelve mobile traps [35] were deployed and baited daily from the 4^th^ June 2013 until the end of August. Each animal was initially identified using numbered ear-tags, and animals captured at the same time and place were marked the same way (colour and shape of tags) as a first indication of the family group. The assignation to family groups was further confirmed using video-cameras on feeding capture sites (such as described by Rossi et al. [36]). Each wild boar was bled at each capture, with a limit of one sampling per week and individual in order to limit an animal’s stress at handling. Ethic and authorization rules were the same as described by Rossi et al. [9, 36]. An intensive information campaign (i.e., using mailings, local newspapers, meetings, telephone calls) was conducted in order to encourage hunters from the whole Vosges du Nord area to notice and sample systematically marked animals from September 2013 up to December 2014.

#### Samples and diagnosis

Serological responses were measured in sera (collected with dry tubes centrifuged in the few hours after capture) and virological examinations were performed on whole blood from captured animals (using tubes with EDTA) or spleen in hunted ones. Sample sera were first tested by an ELISA (Idexx CSF Ab) according to the manufacturer’s instructions. Then, positive or doubtful samples were analyzed by differential VNT. Two CSF virus strains were considered here for VNT: the Bas-Rhin strain (i.e., the wild type of the virus isolated from 2003 up to 2007 in the study area, [21]) and the Alfort strain (i.e., genetically related with the vaccine C-strain). RNA was purified from whole bood using the Rneasy minikit (Quiagen, Courtabeuf, France). The CSF genome was first amplified by real-time polymerase chain reaction (r-RT-PCR) using a commercial kit (LSI VetMAX™ classical swine fever by LSI-Life technologie or Adiavet CSF Real Time by Adiagene-Biomerieux) according to the manufacturer’s instructions. To confirm that piglets were viremic at the time of capture (i.e., carrying viral particles in their blood), virus isolation was performed on the PCR positive samples, on PK15 cells, according to the OIE’s manual of diagnostic tests [25].

## Results

### Surveillance of hunted wild boar

#### Raw serological data

**Figure 2:**
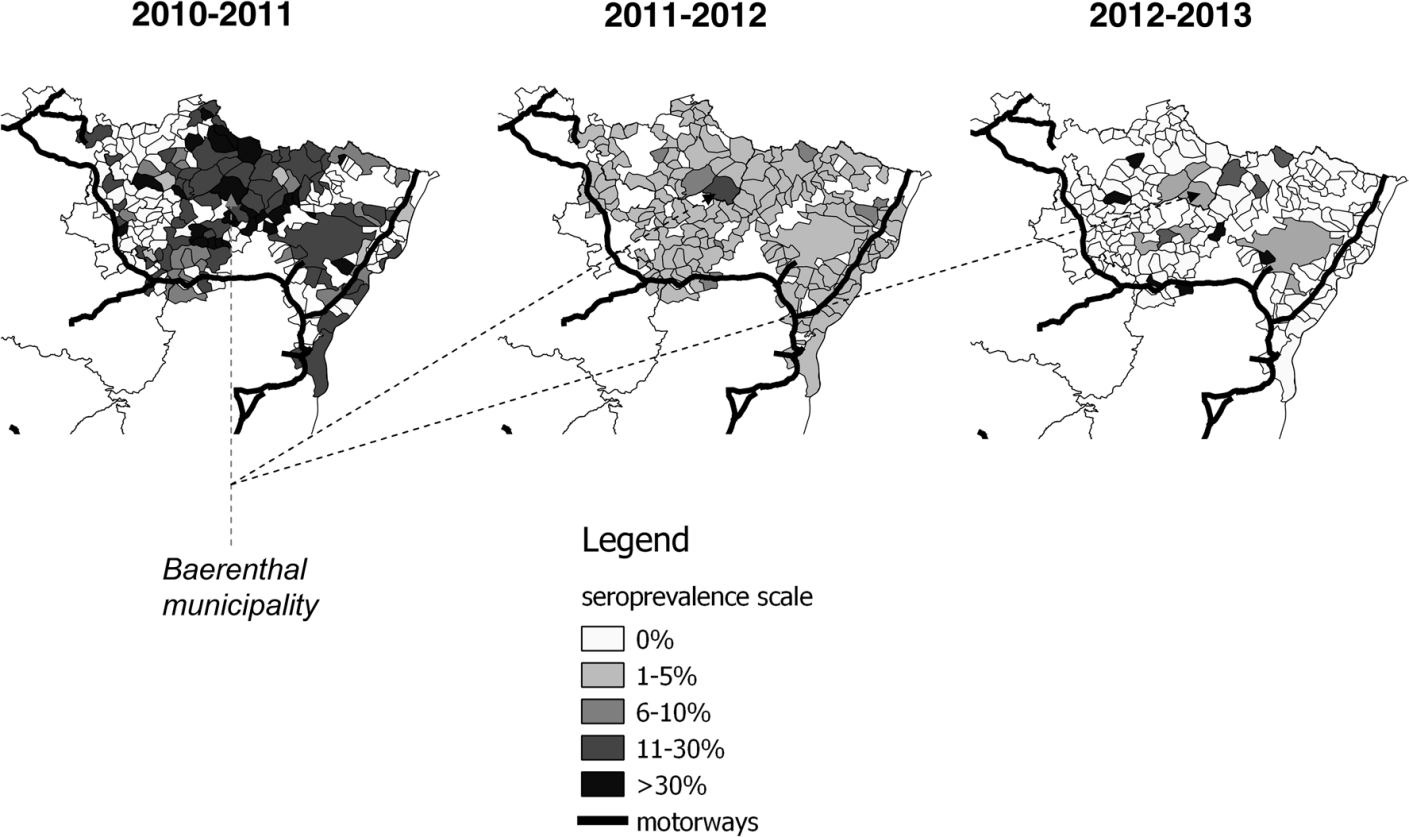
**Maps of the raw CSF seroprevalence observed per municipality and per year.** The grey scale represents raw seroprevalence per municipality.

From July 2010 to June 2013, 9331 6–12 month-old hunted wild boars were sampled and exhibited a conclusive serological ELISA result. On average, the seroprevalence dramatically decreased from 27.8% in July–September 2010 (±1.0%) down to 1% in April–June 2013 (±0.8%). However, raw seroprevalence remained high (i.e., with local peaks above 5%) at the level of some municipalities (Figure 2). In 2012–2013, 46 out of 56 (~80%) sera having a positive doubtful result to the ELISA and subsequently tested by VNT had detectable levels of neutralizing antibodies targeting specifically the CSF not the BD virus. The remaining 20% sera with ELISA positive or doubtful results exhibited undetectable levels of neutralizing antibodies against both CSF and BD viruses, possibly as a result of low CSF antibody titres or unspecific ELISA results.

#### Spatial patterns of seroprevalence in young wild boar

**Figure 3:**
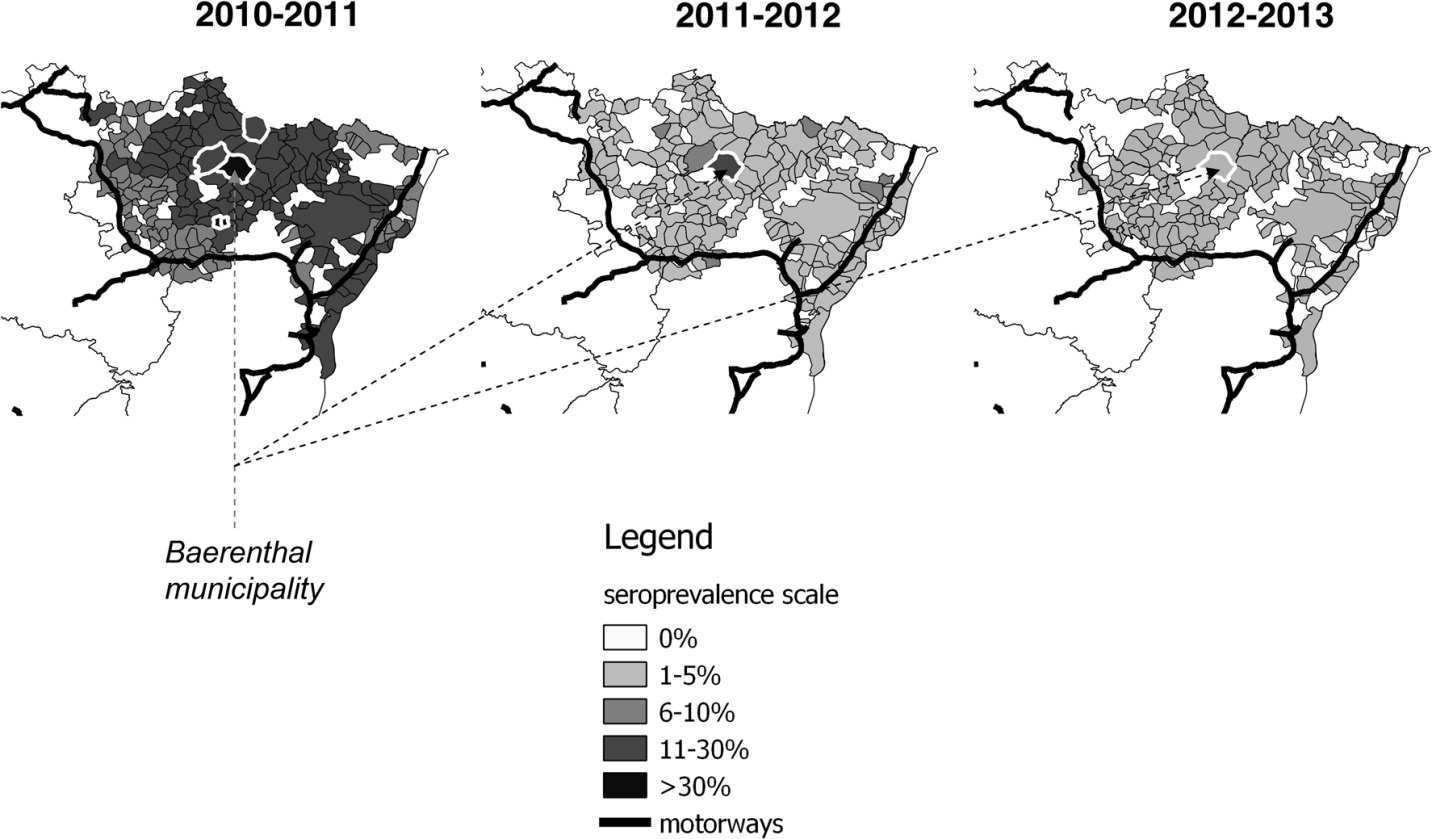
**Maps of the predicted CSF seroprevalence per municipality and per year.** The grey scale represents predicted seroprevalence per municipality and the white surrounded municipalities correspond to a higher seroprevalence compared to the average seroprevalence.

Modelling was based on 3654 conclusive serological results collected from October 2010 to June 2011, 2387 collected from October 2011 to June 2012, and 2840 collected from October 2012 to June 2013. Sample size ranged from 14 up to 345 depending on the municipality and the year. The tested models for each year and corresponding DIC values are detailed in Table 1. During the whole study period, the model best fitting the observed data included the local autocorrelation component “*U*_*i*_” but not the global one “*H*_*i*_” (i.e., the DIC of model M3 was lower than the DIC of model M4 in Table 1). In 2012–2013, the best model also retained the effect of the seroprevalence predicted by the model of the previous year “*Risk*_*i*_” (i.e., the DIC of model M5 was the lowest in Table 1). We represented the predicted seroprevalence according to the best model for each year (Figure 3). According to these predictions, only some municipalities exhibited higher seroprevalence compared to the average seroprevalence (i.e., white surrounded municipalities in Figure 3). The Baerenthal municipality exhibited a higher risk compared to other municipalities during the whole study period (Figure 3).

**Table 1:** **Spatial models and their DIC.**

Model name	Explicative variables	2010–2011	2011–2012	2012–2013
**M1**	**b**_**0**_	DIC = 635.6	DIC = 210.5	DIC = 113.1
**M2**	**b**_**0**_ **+** **H**_**i**_	DIC = 543.7	DIC = 155.7	DIC = 112.1
**M3**	**b**_**0**_ **+** **U**_**i**_	***DIC = *** ***278.7***	***DIC = *** ***39.0***	*DIC* = *110.2*
**M4**	**b**_**0**_ ** +** **U**_**i**_ ** +** **H**_**i**_	DIC = 289.0	DIC = 155.2	DIC = 111.4
**M5**	**b**_**0**_ **+** **U**_**i**_ **+** **b**_**1**_^**a**^ **Risk**_*i***(N-1**)_	Not available^a^	DIC = 150.7	***DIC =*** ***105.2*** ***b***_*1*_ **=** ***0.36, [0.05; 0.61]***

U_i_ and H_i_ correspond respectively to the local and to the global spatial components, Risk_i_ corresponds to the risk predicted the previous year at the level of each municipality (i). Parameter value (b_1_) is indicated together with its credibility interval at the risk of 95% [(2.5%; 97.5%)]. The model retained each year (according to the DIC) is indicated in bold and italic

^a^The study started by 2010–2011, i.e. after the completion of vaccination

### Capture-mark-recapture study

#### Sample of captured animals

From the 2^nd^ of July up to the 30^th^ of August 2013, 134 wild boars were captured and marked comprising 107 piglets, 19 subadults and 8 adults. During the capture process, 94/134 (70%) were captured once, 25/134 (19%) twice, 11/134 (8%) three times and 4/134 (3%) four times. Video-cameras collected about 39 800 pictures corresponding to 893 wild boar visits of the 12 trapping sites, allowing the identification of 25 social groups: 14 groups comprising adult females and piglets, nine groups comprising only subadults of both sexes, one single adult sow without piglet, and one single adult male. From the 23^rd^ July 2013 up to the 20^th^ December 2014, 51/134 marked animals were found at death time (38%): 47/51 had been shot during hunting, 2/51 had been found sick, and 2/51 had been found dead after a road accident. Dead animals comprised 2/8 adults, 12/19 subadults and 37/104 piglets. The place of death was confirmed for 49/51 individuals: 38/49 in the municipality of capture, 9/49 in a neighbouring municipality (i.e., less than 5 km from their capture site) and 2/49 up to 10 km from their captured site.

#### Serological and virological results

None of the captured wild boar were found positive by rt-RT-PCR but 12/134 animals exhibited at least one positive ELISA result: 11 (11/107) piglets belonging to five groups and one adult sow older than 30 months old belonging to the same group as the three seropositive piglets (Table 2). The two sick individuals who died during summer 2013 were necrospsied and exhibited general weakness associated with respiratory distress and important worm loads (genus *Metastrogylus*) in the lungs (bronchial tubes), but they were both negative by serology and PCR. The kinetics of antibodies among the twelve seropositive individuals is detailed in Table 2. The neutralizing antibody titres observed in piglets were low (20) compared to the adult sow (360). Among the piglets recaptured from July to August 2013, either ELISA results became negative or VNT antibody titres decreased. Among the remaining piglets, all but one could be sampled during hunting from which only one, shot the 10^th^ November 2013, showed a positive ELISA result at around 6–7 months and a low neutralizing antibody titre (7.5). No other seropositive result was reported in the marked animals until December 2014 (Table 2).

**Table 2:** **Detailed antibody kinetics of seropositive captured wild boars.**

Individual features				Animals live-captured from 2nd July up to 30th August 2013 (number of the week)								**Animals shot during hunting**				**total**
Groupe code	Tag	age	sexe	27	28	29	31	32	33	34	**35**	**46**	**47**	**52**	29*	
G1	58	Piglet	M	*ELISA*+ *VNT*+ *(10; 7.5)*				ELISA -	ELISA-	ELISA-						4
	61	Piglet	M	*ELISA*+ *VNT*+ *(15; 10)*				ELISA -	ELISA-							3
G15a	125	Piglet	M				*ELISA*+ VNT- (7.5; <5)	*ELISA*+ VNT- (5; 5)						ELISA-		3
	126	Piglet	F				*ELISA*+ VNT- (7.5; <5)							ELISA-		2
G15b	76	Piglet	M		*ELISA*+ *VNT*+ *(40; 7,5)*						ELISA-					2
	94	Piglet	F			ELISA doub. *VNT*+ *(15; 7,5)*					ELISA-		ELISA-			3
	99	Piglet	F			ELISA doub. *VNT*+ *(15; 10)*					ELISA-					2
	132	Piglet	M					*ELISA*+ Inconclusive VNT						ELISA-		2
	289	Adult	F		*ELISA*+ *VNT*+ *(320; 160)*											1
G19	123	Piglet	M				Serum not available					***ELISA*** **+** VNT- (7.5;7.5)				1
G6	72	Piglet	F	*ELISA*+ *VNT*+ *(10; 10)*												1
	74	Piglet	M	*ELISA*+ *VNT*+ *(7,5; 10)*											ELISA-	2

VNT were realised when the ELISA test was positive or doubtfull, positive results are indicated in bold and italic. Neutreulizing antibodies titers for the two tested virus strains are indicated in parentheses (Bas-Rhin strain; Alfort strain)

## Discussion

At a global level, the average seroprevalence observed in 6–12 month-old hunted wild boars has continuously decreased during the 3 years following the completion of OMV. However, at a local level, seroprevalence peaks above 5% raised the question of a residual circulation of CSF or related virus. Most of ELISA positive results were associated with detectable titres of CSF neutralizing antibodies (~80%), thus confirming that positive ELISA results were corresponding to antibodies targeting the CSF virus. A study conducted at two different spatial levels (i.e., inter and intra-municipalities) was adopted to clarify the situation.

At the inter-municipality level, the hierarchical Bayesian approach was useful before mapping seroprevalence [27]: maps predicted by the models (Figure 3) were indeed quite different from the raw seroprevalence ones (Figure 2). The spatial modelling of hunting data accounted for an important autocorrelation of seroprevalence between adjacent municipalities at the scale of the whole area (i.e., the local spatial component “*U*_*i*_” led to a dramatic decrease in models’ DIC), especially during the two first years. Autocorrelation could correspond to a contagious phenomenon, i.e. the active circulation of CSF virus between municipalities. The capture study showed that a significant proportion of wild boars captured in a given municipality were shot elsewhere (~23%), mainly in the neighbouring municipalities. The low match between municipalities’ boundaries of and wild boar home ranges could also participate in the autocorrelation of seroprevalence. We did not detect an important heterogeneity at a global scale (i.e., the global spatial component “*H*_*i*_” led to no improvement in models’ DIC). Nevertheless, some isolated municipalities located in the heart of the forested area, exhibited a higher seroprevalence compared to others and this spatial structure was particularly conserved from 2011–2012 to 2012–2013 (i.e., the Baerenthal municipality being more at risk during the 3 years of the study). Identifying seroprevalence hot spots was interesting for targeting the surveillance efforts, but could not answer the question of the origin of antibodies in 6–12 month-old wild boars (i.e., CSF infection, vaccination, persistence of maternal antibodies). We thus used a longitudinal approach at a finer scale for clarifying the epidemiological situation.

The capture-mark-recapture performed at the level of the seroprevalence hot spot (i.e., Baerenthal and neighbouring municipalities) showed no virus positive animal. This result could either correspond to CSF eradication or to the low number of sampled wild boar (134 individuals allowing the detection of about 3% of viroprevalence), we thus looked at the semi-quantitative serological results. During July–August 2013, about 10% of the 3–5 month-old piglets, and one out of the seven adult sows above 30 months old (i.e., possibly born before the completion of vaccination) were found seropositive. Seropositive piglets had much lower neutralizing antibody titres than the seropositive adult sow, and they progressively lost their antibodies from July 2013 to November 2013 (Table 2). A single individual out of eleven piglets seropositive when 3 months-old was still seropositive during hunting, i.e., when 6–7 months-old. This pattern was consistent with previous experiments conducted in pigs or wild boar showing maternal antibody transmission by immune sows to their offspring several years after challenge of infection [15], and the rare occurrence of long-lasting maternal antibodies in animals after their 6^th^ month [17]. An active immunization of piglets, caused by persisting natural infection, would have induced higher titres of neutralizing antibodies and the subsequent persistence of antibodies [37]. Our capture-mark-recapture study thus suggests that the local peaks of seroprevalence (>5%) observed at the level of municipalities was generated by the survival of immunized sows more than 3 years after the completion of vaccination. The detection of antibodies in young animals above 6 months of age possibly occurred after some seropositive sows were repeatedly vaccinated before June 2010 and conserved high antibodies titres many years after the last vaccination campaign. In that area about 600 000 vaccine-baits had been delivered per year for 6 years [6, 16] and vaccine were mainly consumed by adult animals [38], so that one may assume that the intensity of the vaccination treatment was the main cause for the presence of high antibodies titres in sow’s offspring. The progressive disappearance of vaccinated sows due to natural mortality and hunting together with the probable decrease of antibody titres from year to year could finally account for the progressive decrease of young seroprevalence over time.

In the present paper we investigated the reason of the presence of seropositive juvenile wild boars after the completion of oral mass vaccination (OMV) combining large scale hunting data and local longitudinal survey on marked animals. Due to autocorrelation in the hunting data, a hierarchical Bayesian approach was implemented to decrypt the spatial structure of seroprevalence. The models revealed the presence of localized hot spots of seroprevalence, at the level of some municipalities. However, only the longitudinal survey of marked individuals from the hot spots areas could disentangle the possible sources of antibodies in 6–12 month-old wild boars. In the present case, the progressive disappearance of antibodies in repeatedly captured piglets suggests that hyper-immunized sows having transmitted maternal antibodies to their offspring could explain seropositivity in young wild boars during the 4 years following the completion of OMV. Our approach offers an objective way to interpret surveillance data and adapt the surveillance process after the completion of OMV. This study also pinpoints the difficulty of interpreting seropositivity in young wild boar post-vaccination by simply examining transversal serological results and qualitative serological results. On the contrary to what was initially expected by European experts [3, 14], the “ghost of vaccination” may haunt the antibodies response of 6–12 months-old wild boars several years after the completion of vaccination, which strongly interacts with the efficacy of surveillance based on hunting data.

In that context, we recommend combining different surveillance approaches post-vaccination. First, the passive surveillance of dead or sick animals should be strengthened (i.e., reinforced collection of carcasses) since CSF re-emergence is supposed to cause morbidity and mortality in naïve populations [3]. Nevertheless, the low probability of carcass detection in dense forested areas and the presence of scavengers sometimes limit the efficacy of passive surveillance for early detection ([23], Rossi, unpublished observations). Thus, we also recommend maintaining an active surveillance based on young wild boar serology combined with data modelling for identifying seroprevalence hot spots above 5%. When such hot spots are detected, we recommend targeted longitudinal surveys using quantitative serology (VNT) and virus genome detection. In the future, the use of marker vaccine and companion tests could also be considered as a possible tool for better disentangling the origin of antibodies during and after vaccination [39].

## Additional file

10.1186/s13567-015-0289-6 **“Disease” mapping model.** This document details the hierarchical spatial Bayesian model computation.

## Abbreviations

BD: border disease
CSF: classical swine fever
OIE: World Organisation for Animal Health
OMV: oral mass vaccination
PCR: polymerase chain reaction
r-RT-PCR: real-time polymerase chain reaction
VNT: virus neutralisation test
