# Supplementary material for: How to survey classical swine fever in wild boar (Sus scrofa) after the completion of oral vaccination? Chasing away the ghost of infection at different spatial scales
Source: Vet Res. 2016 Jan 25;47:21. doi: 10.1186/s13567-015-0289-6 (PMC4727256; doi:10.1186/s13567-015-0289-6)
Supplement: Supplementary file 1 — 10.1186/s13567-015-0289-6 “Disease” mapping model. This document details the hierarchical spatial Bayesian model computation. [file 13567_2015_289_MOESM1_ESM.doc]

## Additional file 1 “disease” mapping model

## Model construction and validation

We studied the relative risk to observe seropositive 6-12 month-old hunted wild boars during a given year within each spatial unit “i” (i.e., French municipalities). The spatial unit “i” is defined as the administrative boundary of each municipality within the Vosges du Nord area. We considered observations were independent and identified no individual or group factor influencing seroprevalence. Assuming a constant risk over space, the expected number of seropositive in each spatial unit should be e_i_=(C_t_/P_t_)*P_i_, C_t_ being the total number of cases, P_t_ the total population size and P_i_ the municipality population size. The effective number of seropositive in each unit is supposed to follow a Poisson distribution with mean lambda_i_, which is the product of expected number of cases with the relative risk RRi: lambda_i_=e_i_*RR_i_.

We fitted five different Bayesian hierarchical models (subsequently named M1 to M5, see below) considering separately the three periods of sampling in order to provide a map of seroprevalence for each generation of wild boar born after vaccination completion. Posterior distributions of parameters were deduced from prior information about parameters and likelihood functions of the data and can be estimated by Monte Carlo Markov Chain (MCMC) simulations, i.e., inferences are made empirically by collecting many realizations from the posterior distribution using a variant of Metropolis method called Gibbs sampling. We ran this sampler for an initial period of 140 000 cycles (burn-in period) and then collected information for the next 100 000 iterations of which we stored every 10 cycles. The stability of the chain was verified with the Heildelberger-Welch test. We first confirmed the presence of effective data autocorelation by inspecting graphically the semi-variogram of observed seroprevalence at the scale of the municipalities. Since overdispersion could invalidate our Poisson model, we also tested the absence of overdispersion comparing the mean and the variance of predictions. Models’ selection was based on the Deviance Information Criterion (DIC): we retained the most parsimonious model having the smaller DIC. Furthermore, we controlled that parameters were significantly different from zero by using credibility intervals. Because we had no prior information about parameters we used flat priors. We used 95% Bayesian credibility intervals corresponding to the 2.5^th^ and the 97.5^th^ percentile of the parameter’s posterior distribution.

## Analysis of the spatial structure

Due to wild boar mobility, we expected non independence of the relative risk between adjacent spatial units. So we first tested four different competitive models:

1. ***A model without any spatial structure: log(RR_i_)=b_0_ (M1)***

This model depicts a constant risk over the whole area. Parameter b_0_ corresponds to the intercept and its prior distribution was uniform.

1. ***A model with a local spatial component U_i_: log(RR_i_)=U_i_+b_0_ (M2)***

This model is known as the Conditional Auto-Regressive model (CAR). We could estimate relative risk while considering spatial interactions between neighbouring municipalities through a local spatial smoothing factor U_i_. It assumes that values of a pair of contiguous municipalities would be generally much more alike than for two arbitrary municipalities. Its prior distribution is Gaussian autoregressive. U_i_~N(Ū_δi_,τ_ui_). Ū_δi_ is the mean of the spatial components among the set “δi” of municipalities adjacent to the municipality “i”. The variance τ_ui_ is inversely weighted by the number of neighbours for each spatial unit. The hyperparameter τ_ui_ is empirically assigned to a gamma prior distribution with shape and scale parameter equal to 0.5 and 0.0005 respectively.

1. ***A model with an unstructured spatial component hi: log(RR_i_)=H_i_+b_0_ (M3)***

The global spatial component H***_i_*** was also introduced for testing heterogeneity of seroprevalence among the municipalities. The prior distribution of H***_i_*** is a realisation of a Gaussian white noise H_i_~N(μ_Hi_,τ_H_) with mean μ_Hi_ and variance τ_H_ (the hyperparameter) is assigned to a gamma prior distribution with shape and scale parameter equal to 0.5 and 0.0005 respectively.

1. ***A model with both Ui and Hi: log(RR_i_)=U_i_+H_i_+b0 (M4)***

## Relative risk prediction the year before: log(RR_i_)=Si+ b_0_+b_1_*Risk_i_ (M5)

Starting from the best spatial model (S***_i_***), we subsequently tested the conservation of the risk structure from year to year. Posterior mean of Relative Risk in spatial unit i calculated for the model at time t-1 (Risk***_i_***) is incorporated in the model at time t. It was not included in the model for the first period. This variable was tested for assessing the conservation of the spatial structure of the risk from year to year. Parameter b_1_ was affected a uniform prior distribution
